# Comparison of Two Diagnostic Scores of Disseminated Intravascular Coagulation in Pregnant Women Admitted to the ICU

**DOI:** 10.1371/journal.pone.0166471

**Published:** 2016-11-18

**Authors:** Marie Jonard, Anne Sophie Ducloy-Bouthors, François Fourrier

**Affiliations:** 1 Department of Intensive Care Unit, Centre Hospitalier du Dr. Schaffner, Lens and Centre hospitalier Roger Salengro, Centre Hospitalier Regional Universitaire, Lille, France; 2 Department of Obstetric Anesthesia, Centre Hospitalier Regional Universitaire, Lille, France; 3 Department of Intensive Care Unit, Centre hospitalier Roger Salengro, Centre Hospitalier Regional Universitaire, Lille, France; Hungarian Academy of Sciences, HUNGARY

## Abstract

**Objective:**

To compare the validity of two previously published diagnostic scores of disseminated intravascular coagulation (DIC) in pregnant women admitted to ICU for an acute thrombotic or hemorrhagic complication of delivery and postpartum.

**Methods:**

This was a population based retrospective study of 154 patients admitted to ICU for severe delivery and postpartum complications in a University Hospital. A recently published score (adapted to physiological changes of pregnancy and based on three components: platelet count, prothrombin time difference and fibrinogen) was compared to the International Society for Thrombosis and Hemostasis (ISTH) score (based on four components: platelet count, fibrinogen, prothrombin time, and fibrin related marker). Both scores were calculated at delivery, ICU admission (day 0), day 1 and day 2 during the postpartum ICU stay. The validity of both scores was assessed by comparison with the consensual and blinded analysis of two experts. The sensitivity, specificity, and area under the curve (AUC) of each score were calculated at each time and overall by generalized linear mixed model. The agreement between the two scores was evaluated by the Kappa coefficient.

**Results:**

The new score had a sensitivity of 0.78, a specificity of 0.97 (p <0.01) and a global AUC of 96% while the ISTH score had a sensitivity of 0.31, a specificity of 0.99 and an AUC of 94% (p <0.01). The Kappa coefficient of correlation between both scores was 0.35. The lower sensitivity of the ISTH score was mainly explained by the lack of fibrinogen and fibrin-related peptides thresholds adapted to the physiological changes of coagulation induced by pregnancy.

**Conclusion:**

The new DIC score seem highly discriminant in the subset of patients admitted to the ICU after delivery for an acute specific complication. The ISTH score is not recommended in pregnant women because of its poor sensitivity.

## Introduction

Pregnancy is associated with physiologic changes of the hemostasis system. To prevent an excessive blood loss during labor and after delivery, a procoagulant imbalance is gradually induced by endothelial activation, increased liver synthesis of coagulation factors, and decreased activity of coagulation inhibitors and fibrinolysis [1]. This fragile dynamic process can swing to a thrombotic state following an additional activation of coagulation or conversely to a severe hemorrhagic syndrome when consumption of hemostasis factors is no longer compensated or fibrinolysis is out of control. A disseminated intravascular coagulation (DIC) can occur in these pathological conditions and is associated with significant maternal and fetal morbidity and mortality. Therefore, a prompt diagnosis and an accurate monitoring of DIC are important to optimize the management of these patients [2].

Interpretation of these rapid and complex coagulation changes is difficult for clinicians. It should be based on reliable, fast, and reproducible parameters. The development of diagnostic scores can bring a great help in these situations. Apart from obstetrical situations, the International Society for Thrombosis and Hemostasis (ISTH) has proposed a DIC score combining, in a determined pathological context, acute changes in hemostasis parameters (platelet count, Prothrombin time (PT), fibrinogen, and D-dimers or fibrin split products) [3]. Many studies have shown a good correlation between this score, the diagnosis of severe DIC, and mortality, especially during severe sepsis and polytrauma [4,5]. However, during pregnancy, physiological hemostatic changes may alter the significant thresholds of this score and compromise its accuracy.

More recently, an Israeli team, using a retrospective analysis of a large database created a new DIC score, specifically dedicated to pregnant women [6]. Among 24,693 deliveries, 87 were complicated with DIC and comprised the study group. The diagnosis of DIC in the database was based upon the clinical diagnosis notified in medical records by the physicians in charge, in case of "severe maternal hemorrhage associated with prolonged PT as well as APTT, and low fibrinogen concentrations that required blood products transfusion". To construct the new score, the authors selected platelet count, fibrinogen concentration, and the PT difference versus control (in seconds), with specific thresholds according to physiological changes of pregnancy. They did not include fibrin related markers (D-dimers and fibrin split products) due to the physiological increase of their concentration during pregnancy [7–9].

To our knowledge, this adapted score has never been validated in a population of pregnant women suffering from severe complications of pregnancy likely to develop thrombotic or bleeding disorders. The main objective of our study was to compare the diagnostic power of this new score to the ISTH one in critically ill pregnant women admitted to ICU for acute specific complications of pregnancy or early postpartum. Secondary objectives were to study the sequential changes of both scores during the early postpartum, and explain their discrepancies.

## Patients and Methods

We performed a retrospective study including over a seven years period (January 2008—December 2014) all women who presented an acute complication at the end of their pregnancy or during delivery and were transferred immediately after childbirth to the intensive care unit (ICU) of our university hospital. The definitions of pregnancy acute complications are given in S1 File. The primary endpoint of the study was to compare the predictive values of the new score to the ISTH one in this population of acutely ill obstetrical patients.

Case notes were retrospectively analyzed. All patients files were anonymized and numbered on a computerized database by a clinical research assistant. Authors didn’t have access to patient identifying information. A approval declaration of non-interventional study was given by our ethics committee and the CNIL (Commission Nationale de l’Informatique et des Libertés) specifically approved this study with the reference DEC14-2. According to French law, informed consent of the patients was no required due to the retrospective, anonymous, and non interventional nature of the study. Medical and biological records of all patients were examined from delivery until the second day of postpartum. Blood samples collected at delivery and at day 0, day 1, and day 2 during the postpartum ICU stay were analyzed in the Laboratory of Biochemistry and Hemostasis of our university hospital. The ISTH score and the new score were calculated at the same times. For comparing their predictive values, we used, as “gold standard”, an expert sequential analysis based on clinical and laboratory data of each patient. Two experts who shared at least a thirty years experience in obstetrical pathology, intensive care, and hemostasis, had to respond to the presence or absence of DIC at each time point, independently. They were blinded to the results of the two scores and asked not to calculate their values. In case of disagreement, they were required to re-analyze the file together to reach a consensus. The diagnostic power of each score was tested in reference to this expert analysis and the different components of both scores were compared between patients classified DIC (+) and DIC (-) to explain their possible discrepancies. Criteria of DIC of the ISTH score and the new score are given in S1 and S2 Figs. All the data are available in the online supplementary files.

## Statistical Analyses

To evaluate the diagnostic power of the two scores in comparison to the expert analysis, their sensitivity, specificity, area under the curve (AUC), and 95% confidence intervals were calculated overall and sequentially from delivery to Day 2. At each time point, we categorized the case as DIC + or DIC—according to the predefined thresholds of the two scores (≥26 for the new one and ≥5 for the ISTH one). Their sensitivity and specificity were calculated and we performed logistic regression to calculate receiver operating characteristics (ROC) and AUC. In the overall analysis, we used a generalized linear mixed model to account for repeated measurements.

The agreement between the two scores (categorized into two classes) was evaluated globally and at different times using the Kappa coefficient of Cohen.

The relationship between the various components of the scores and expert analysis was studied by the chi-square or Fisher exact tests for different times and by a generalized linear mixed model for the overall analysis.

Statistical analyses were realized by the Biostatistics and Methodology Unit of our University Hospital (Pr. A. Duhamel, J. Labreuche, E. Drumez) and used the SAS software (Version 9.4). The level of significance was set at 5%.

## Results

Over the 7-year study period, 185 patients were admitted to our ICU for acute complications occurring at the end of pregnancy or during delivery. 154 patients were included in the study and 31 patients were excluded: 15 patients admitted during pregnancy for acute respiratory failure, severe early preeclampsia or sickle cell disease; eight admitted postpartum for a chronic disease; one for late postpartum thrombotic thrombocytopenic purpura. Seven medical observations were incomplete.

The two main complications triggering admission to the ICU were severe toxemic syndromes and postpartum hemorrhage. Fifty-seven percent of patients (n = 88) suffered from toxemia, with 27 cases of preeclampsia and 61 cases of HELLP syndrome. Eclampsia occurred in 22% of these patients (n = 19). Fifty-five per cent of the patients (n = 85) developed postpartum hemorrhage (PPH), with five cases among 85 due to placental abruption. PPH was associated with preeclampsia in 7% of cases (n = 6) and HELLP syndrome in 29% (n = 25). Six cases of acute fatty liver of pregnancy were identified. Main characteristics are outlined in Table 1 and S1 Table.

**Table 1:** Characteristics of all patients with and without DIC.

		Population globale n = 154
**Age**^**x**^ **(years)**		30 (26–35)
Number of pregnancy*		2 (1–3)
Parity*		2 (1–3)
Term (week of gestation)*		36 (31–39)
**Mode of delivery** ^**x**^^,^^**a**^:		
	**Vaginal childbirth**	47 (30)
	**Cesarean delivery**	106 (69)
**Type of anesthesia** ^**x**^^,^^**b**^** :**		
	**Regional anesthesia**	77 (50)
	**General anesthesia**	63 (41)
Length of stay in the ICU (days)*		3 (2–5)
**Diagnosis** ^**x**^		
	**PE**	27 (17)
	**HELLP syndrome**	61 (40)
	**Eclampsia**	19 (12)
	**AFLP**	6 (4)
	**PPH**	85 (55)
	**Sickle cell disease**	1
	**Thrombotic Microangiopathy**	1
	**Septic Shock**	1
	**Cerebral venous thrombosis**	1
	**Hemorragic shock (traumatic)**	1
	**Anesthesia complications**	1
SAPS II score*		20 (13–29)
**Fetal death** ^**x**^^,^ ^**c**^		19 (12)
**Complications**		
	**Hemodynamic Instability**^**x**^	34 (22)
	**Sepsis**	16 (10)
**Acute renal failure**^**x**^		63 (41)
**RRT**^**x**^		14 (9)

^X^ Data are N (%) for qualitative variables

DIC: disseminated intravascular coagulation. PPH: Post Partum Hemorrhage. PE: Preeclampsia. HELLP syndrome: Hemolysis, Elevated Liver enzymes, Low platelet count Syndrome. AFLP: Acute Fatty Liver pregnancy. SAPS II: simplified acute physiological score. RRT: Renal Replacement Therapy

*Quantitative variables are presented as median [interquartile range: 25th to 75th percentiles]

^a^ 1 missing data

^b^ 14 missing data

^c^ 2 missing data

### Diagnostic power of the two scores (Fig 1 and Table 2)

The diagnosis of DIC according to the "expert gold standard" was made in 45% of patients at delivery and 55% at admission in ICU, respectively (S2 Table).

**Fig 1:**
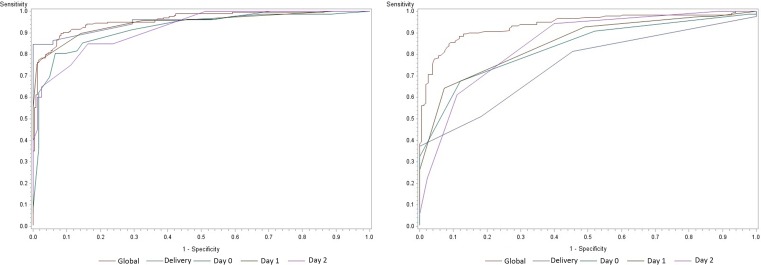
ROC (Receiver operating characteristics) curve analysis of the sequential diagnostic power of the two scores. The Curve analysis of each day: day of delivery (Delivery), Day 0, Day 1, Day 2 and curve analysis of the sum of all measured values (Global). The figure on the left represents the ROC curves of the new DIC score. The figure on the right represents the ROC curves of the ISTH score.

**Table 2:** Receiver operating characteristics of the two scoring systems.

Variable	Se	Sp	AUC	95% confidence interval		p
				Lower	Upper	
**New score**						
Delivery (n = 69)	0.84	1	0.95	0.91	0.99	<0.01
Day 0 (n = 143)	0.80	0.93	0.92	0.87	0.96	<0.01
Day 1 (n = 134)	0.75	0.98	0.95	0.90	0.99	<0.01
Day 2 (n = 100)	0.60	0.98	0.92	0.85	0.98	<0.01
Global (n = 446)	0.78	0.97	0.96	0.94	0.98	<0.01
**ISTH score**						
Delivery (n = 54)	0.37	1	0.75	0.61	0.89	<0.01
Day 0 (n = 127)	0.32	1	0.83	0.77	0.90	<0.01
Day 1 (n = 97)	0.26	1	0.85	0.78	0.92	<0.01
Day 2 (n = 63)	0.22	0.98	0.85	0.76	0.94	<0.01
Global (n = 275)	0.31	0.99	0.94	0.91	0.96	<0.01

AUC: area under the curve; ISTH: International Society on Thrombosis and Haemostasis. Se: sensitivity; Sp: specificity

Regarding the predictive power of the new score, the overall AUC was 96%. As globally calculated from the sum of all measured values, the sensitivity of the new score was 0.78, and its specificity reached 0.97 (S3 Fig). During the ICU stay, sensitivities of the score progressively decreased over time: 0.84 at delivery, 0.80 at ICU admission, 0.75 at day 1, and 0.60 at day 2. In all cases, specificity was higher than sensitivity (Table 2).

Regarding the predictive power of the ISTH score, the overall AUC was 94% with a good specificity (0.99) but low sensitivity (0.31) (S3 Fig) and AUC at each time point. The sensitivity decreased over time while the specificity remained high (Table 2).

The agreement between the two scores was 71%, with a Kappa coefficient of 0.35 (S3 Table).

#### Influence of the cut-off levels on the diagnostic power of each score

Concerning the new score (Table 3), the cut-offs chosen by Erez et al for PT difference and fibrinogen were consistent with our expert analysis. When the PT difference was ≥ 1sec (giving 12 points at least in the score), DIC was found in more than 70% of cases according to expert opinion. DIC was found in less than 50% of cases when the PT difference was < 1 sec. When the fibrinogen concentration was lower than 3g/l (giving 25 points in the score), DIC was found by expert analysis in more than 90% of cases. The platelet threshold seemed less suited (see table). A diagnosis of DIC was given by the experts in 70% of cases when platelet count was lower than 50.10^9^/L. However, when platelet count was between 50 and 100.10^9^/l, the diagnosis of DIC was given by the experts in 46% of cases only, whereas the new score gives the maximum of 2 points within these limits.

**Table 3:** Number and percentage of patients classified as DIC+ by expert analysis and their repartition according to the cut-offs of the three components of the new DIC score.

	DIC + (by experts)				
Time	Delivery	Day 0	Day 1	Day 2	Global
**PT difference (sec)**					
**<0.5**	13/31 (42%)	26/77 (34%)	22/102 (21%)	7/83 (8%)	68/293 (23%)
**0.5–1**	6/7 (86%)	3/8 (37%)	4/8 (50%)	1/3 (33%)	14/26 (54%)
**1–1.5**	7/7 (100%)	18/23 (78%)	7/10 (70%)	2/3 (67%)	34/43 (79%)
**>1.5**	26/26 (100%)	35/36 (97%)	16/16 (100%)	10/11 (91%)	87/89 (98%)
**p**	<0.01	<0.01	<0.01	NA	<0.01
**Platelet count (x.10**^**9**^**/L)**					
**<50**	10/12(83%)	24/31 (77%)	17/21 (81%)	3/11 (27%)	54/75 (72%)
**50–100**	20/24 (83%)	31/53 (58%)	15/50 (30%)	11/41 (27%)	77/168 (46%)
**100–185**	30/48 (62%)	28/47 (60%)	15/54 (28%)	4/39 (10%)	77/188 (41%)
**>185**	10/27 (37%)	2/21 (9%)	2/19 (10%)	4/16 (25%)	18/83 (22%)
**p**	<0.01	<0.01	<0.01	NA	<0.01
**Fibrinogen (g/l)**					
**<3**	58/61 (95%)	65/71 (92%)	35/37 (95%)	10/10(100%)	168/179 (94%)
**3–4**	6/15 (40%)	15/36 (42%)	9/31 (29%)	4/16 (25%)	34/98 (35%)
**4–4.5**	2/11 (18%)	3/21 (14%)	2/27 (7%)	3/21 (14%)	10/80 (12%)
**>4.5**	3/18 (17%)	2/23 (9%)	3/50 (6%)	5/60 (8%)	13/15 (19%)
**p**	<0.01	<0.01	<0.01	<0.01	<0.01

Data (%) represents the number of patients DIC + by experts in the cut-off of components. PT: Prothrombin Time. NA: Not Applicable (insufficient number)

Regarding the ISTH score (Table 4), there was a strong discrepancy with the expert analysis. A significant proportion of DIC was found by the experts when the ISTH score gave no point: DIC was found in 60% of cases when the fibrinogen concentration was > 1g/l, and in 98% of cases when PT was between 3 and 6 seconds.

**Table 4:** Number and percentage of patients classified as DIC+ by expert analysis and their repartition according to the cut-offs of the four components of the ISTH score.

ISTH Score	DIC + (by experts)					
	Delivery		Day 0	Day 1	Day 2	Global
**Platelet count (x.10**^**9**^**/L)**						
**<50**	10/12 (83%)		24/31 (77%)	17/21 (81%)	3/11 (27%)	54/75 (72%)
**50–100**	19/23 (83%)		31/52 (60%)	14/49 (29%)	11/39 (28%)	75/163 (46%)
**>100**	40/75 (53%)		30/68 (44%)	17/73 (23%)	8/55 (15%)	95/271 (35%)
**p**	0.01		<0.01	<0.01	NA	<0.01
**FDP**						
**0**		2/4 (50%)	5/24 (21%)	5/42 (12%)	5/34 (15%)	17/104 (16%)
**1**		13/17 (76%)	38/64 (59%)	24/43 (56%)	9/21 (43%)	84/145 (58%)
**2**		18/18(100%)	30/30 (100%)	12/12 (100%)	5/5 (100%)	65/65 (100%)
**p**		NA	<0.01	<0.01	NA	<0.01
**PT difference vs control (sec)**						
**> 6 sec**	8/8 (100%)		5/5 (100%)	5/6 (83%)	2/2 (100%)	20/21 (95%)
**3–6**	21/21(100%)		23/24 (96%)	10/10 (100%)	8/8 (100%)	62/63 (98%)
**< 3**	39/80 (49%)		57/123 (46%)	34/129(26%)	12/97(12%)	142/429(33%)
**p**	<0.01		NA	<0.01	NA	<0.01
**Fibrinogen (g/l)**						
**<1**	15/15 (100%)		6/6 (100%)	2/2 (100%)	2/2 (100%)	25/25 (100%)
**≥1**	54/90 (60%)		79/145 (54%)	47/143 (33%)	20/105(19%)	200/483 (41%)
**p**	<0.01		NA	NA	NA	<0.01

Data (%) represents the number of patients DIC + by experts in the cut-off of components. ISTH: International Society on Thrombosis and Haemostasis. PT: Prothrombin Time. Fibrin/ Fibrinogen Degradation Products: 0 = D-dimers <0.5mg/l or MF<6 μg/ml; 1 = D-dimers 0.5-10mg/l or MF 6–50 μg/ml; 2 = D-dimers >10mg/l or MF >50 μg/ml. NA: Not Applicable (insufficient number)

Regarding platelet count, the expert analysis found 83% of DIC when it was between 50 and 100.10^9^/l at delivery, but this proportion decreased over time. A similar observation was highlighted when there was a moderate increase in fibrinolysis parameters, scoring one point. In this score bracket, the proportion of DIC was high (76%) at delivery but decreased overtime suggesting that a moderate increase in fibrinolysis parameters was not always associated with a pathological fibrinolysis according to expert opinion.

#### DIC according to the pregnancy pathology

(S4 and S5 Tables) The frequency of DIC was significantly higher in the PPH group. Most cases of DIC were relied to the presence of HELLP syndrome complicated by PPH. Among the eight cases of placental abruption, five were complicated by DIC. All six cases of AFLP had DIC according the expert opinion and the new DIC score.

## Discussion

The diagnosis of DIC in pregnant women poses three major difficulties: the lack of a universal definition; the opposite diversity of clinical events (hemorrhagic, thrombotic); the constant coagulation activation at delivery and postpartum. In this situation, a DIC score could bring help, should be easy applicable, enable an early diagnosis by any clinician not expert in this field, and permit a common definition of obstetrical DIC.

The frequency of DIC during pregnancy varies between studies from 0.03% to 0.35% of all deliveries [2,6]. A much higher frequency is observed in patients admitted to ICU, as in our study, where it reached 50%. Taking into account physiological changes in hemostasis of pregnant women, three studies tried to establish a DIC score adapted to this specific population. Terao et al in 1987 has built a score with three components: the underlying pathology, clinical manifestations and laboratory tests based on PT, fibrinogen, FDP and platelet count [10]. However, the low number of patients (n = 77) and the lack of comparison of biological tests with a normal obstetrical population did not allow its validation. Windsperger et al. studied the fibrinogen/C-reactive protein (CRP) ratio to predict overt DIC in patients with HELLP syndrome [11]. This ratio seems to be a better indicator of DIC than the sole fibrinogen level but the peculiarity of HELLP syndrome precludes the use of this ratio in the entire obstetrical population. In the study of Erez et al, the clinical diagnosis of DIC was restricted to deliveries complicated by hemorrhages and based upon the individual reporting by clinicians in charge. In this subset, a score equal to or greater than 26 determined with a high probability the presence of DIC, with an AUC of 97%, a sensitivity of 88%, and a specificity of 96% [6]. In our study, all women suffering from thrombotic or hemorrhagic complications of delivery and late pregnancy were considered at risk of DIC and included in the analysis. The implementation of the new DIC score in our cohort of severely ill patients had a good sensitivity (0.78) and specificity (0.97) with very high AUC. Its diagnostic power was generally better than the ISTH score, which had a good specificity but low sensitivity. The ISTH score does not take into account physiological hemostasis changes occurring in obstetrical situations in contrast to the experts and the new score adapted by Erez. Our study confirms the better predictive value of the new score compared to the ISTH score, with a weak coefficient agreement between both scores (kappa: 0.35).

One objective of our study was to explain differences in the predictive power of the two scores. The diagnosis of DIC by the ISTH score is based on the presence of fibrinolysis products associated with thrombocytopenia and high consumption of clotting factors. The new score focuses on PT difference and fibrinogen with different cut-offs points, a lower influence of thrombocytopenia, and complete exclusion of fibrinolysis parameters.

In our study, when the PT difference was > 1 second, there was a good agreement with the expert analysis (79–98% of DIC). Out of pregnancy, diagnosis of DIC is often considered when PT difference is substantially longer (> 3 seconds in the ISTH score) [3]. However, the physiological increase in coagulation factors in normal pregnancy lowers the PT difference in case of DIC [6,12]. The difference can be even smaller in toxemic syndromes where the “hypercoagulable” state is strengthened [13]. This concept of relative changes justifies a particular attention to PT differences lower than those permitting the usual diagnosis of DIC.

Concerning fibrinogen, the diagnosis of DIC was given by the expert analysis in 90% of cases when its concentration was lower than 3 g/l and reached 100% when the threshold was ≤ 1 g/l, as in the ISTH score. However, in our study, the number of patients with a fibrinogen level < 1 g/l was very low. Consistent with this finding, Bakhtiari et al questioned the interest of fibrinogen concentration in the ISTH score, since such a low level was found in 5% of DIC only [14]. Except in case of massive bleeding or in acute fatty liver of pregnancy, the physiological increase of fibrinogen during pregnancy (4–6 g/l) compared to a general population (2–4 g/l), rarely gives situations where it could decrease to such a low level [7]. Moreover, Charbit et al could demonstrate in women suffering from uterine atony that a fibrinogen concentration < 2 g/l had a positive predictive value of 100% for progression to severe postpartum hemorrhage, whilst a level > 4 g/L had a negative predictive value of 79% [15]. For these reasons, a great importance should be given to the decrease in the level rather than to its absolute value [16]. In comparison to other coagulation factors, any reduction below the high levels found during pregnancy should draw attention. Overall, the specific thresholds of PT difference and fibrinogen for the diagnosis of DIC explain the best concordance of the new score with the expert analysis.

Concerning platelet count, a profound thrombocytopenia (<50,10^9^/l) remains an important point of detection of DIC in the ISTH score unlike the new score. This discrepancy is explained by the presence in Erez’s study of a high number of severely thrombocytopenic patients not suffering from DIC (personal communication). It was probably the same in our population for moderate thrombocytopenia (between 50 and 100 10^9^/ L). This finding suggests that when the other parameters (PT difference, fibrinogen) were normalized, the persistence of thrombocytopenia has been attributed to another cause than DIC by the expert analysis. Accordingly, in the obstetrical setting, the association of thrombocytopenia with DIC should consider more the underlying pathological context and its sequential changes than its absolute level.

Fibrinolysis is decreased during pregnancy due to higher endothelial and placental synthesis of PAI-1 and 2. This situation is totally reversed in the immediate postpartum when occurs an abrupt fall of PAI-2 and Thrombin-Activatable Fibrinolysis Inhibitor (TAFI) of placental origin and the release of plasminogen activators [17,18]. In these conditions, any abnormal activation of hemostasis may be followed by an explosive fibrinolysis affecting both fibrin and plasma fibrinogen [19,20]. In our study we found a high frequency of DIC when D-dimers or fibrin monomers were at the highest (100% of cases). Conversely, at lower levels, the diagnosis of DIC was not always carried (58% of cases). This finding is explained by the occurrence of a moderate physiological fibrinolysis in some obstetrical diseases. Thus, the interpretation of D-dimer and fibrin monomers should remain based on clinical context. The soluble fibrin monomer level offers in theory a superior benefit during DIC since it reflects the action of thrombin on fibrinogen. Soluble fibrin is only produced in the vascular lumen, thus its level is not influenced by extravascular fibrin formation as during local inflammation or trauma. However, most studies have shown a sensitivity of 90 to 100% of soluble fibrin in the diagnosis of DIC but with a very low specificity. It would be useful to evaluate the diagnostic value of this fibrinolysis parameter in the obstetrical population.

### Evolution of DIC

The decrease in the diagnostic parameters of both DIC scores during the stay at ICU reflects the improvement of these patients due to their treatment and the resolution of the acute phase of DIC.

Sequential analysis showed that the prevalence of DIC rapidly decreases in bleeding patients (80% of DIC at delivery, 20% at day 2 according to the expert analysis and the new score) while DIC improved at a lower speed in non-bleeding patients. (30 to 40% of DIC at delivery, 20% at day 2). The correction of DIC and the coagulation rebound occurred earlier (about day 2) in PPH cases provided that mechanical hemostasis is achieved. This contrasted significantly to specific pregnancy pathologies whose coagulation disorders were slower to improve (about day 4 or more for AFLP).

This delay to recover can be exeplained in the 6 cases of AFLP by the association of DIC induced factors and inhibitors consumption to the lack of hepatic regeneration. In PPH, because of the rapid recovery of DIC and postpartum inflammatory syndrome, our data confirm the need of anearly preventive thromboprophylaxis.

Our results give confirmation that severe obstetrical pathologies induce various DIC profiles and sequential changes. In these patients, the hemostatic condition is weak, variable and repetition of hemostasis tests is essential. The ISTH score lacks of sensitivity due to insufficiently adapted thresholds. The new score appears more reliable for DIC monitoring and diagnostic.

### Limitations and Perspectives

Our study has clear limitations. In the study from Erez et al., the new score was established by retrospective extraction from a large database, the diagnosis of DIC being simply notified in patients' records by the physicians in charge, in case of severe hemorrhages. In our study, the “gold standard” diagnosis of DIC was established by a retrospective analysis of patients' data by two experts. Additionally, our study was restricted to severely ill patients admitted to ICU after delivery, with a very high prevalence of DIC, either thrombotic or hemorrhagic. From this point of view, our study should only be considered as the first external evaluation in the ICU setting of a DIC score adapted to physiological changes of pregnancy. Due to missing data after Day 2, our analysis was restricted to delivery and early postpartum, so that it cannot be applied to other stages of pregnancy. Finally our study was not designed to measure the ability of a DIC score to improve the prognosis of these acute specific complications of late pregnancy and postpartum. This could only be done by a prospective multicenter study. Nevertheless, by using a score adapted to physiological changes of pregnancy, an early diagnosis of DIC is likely to reduce the morbidity of these complications.

## Conclusion

Due to its poor sensitivity, the ISTH score should no more be used in pregnant women. The new scoring system adapted to physiological changes of pregnancy seems highly discriminant. Its use will enable non-expert clinicians to establish an early diagnosis of DIC and follow its evolution

## Supporting Information

S1 File Definitions. (DOCX)

S1 Fig New DIC score by Erez. If score ≥ 26: compatible with DIC.
(DOCX)

S2 Fig ISTH DIC score. If ≥ 5: compatible with overt DIC: repeat score daily. If ≤ 5: suggestive (not affirmative) for non-overt DIC: repeat next 1–2 days.
(DOCX)

S3 Fig ROC curve analysis of the global diagnostic power of the new score (DIC) and ISTH score. (TIFF)

S1 Table Characteristics of patients with postpartum hemorrhage. Data are N (%) for qualitative variables. * Quantitative variables are presented as median [interquartile range: 25th to 75th percentiles].
(DOCX)

S2 Table Number and percentages of patients with DIC according to the two scores and expert analysis. Data are N (%) for qualitative variables.
(DOCX)

S3 Table Agreement between the two scores. Data are number; ISTH, International Society of Thrombosis and Haemostasis.
(DOCX)

S4 Table Comparison of the rate of DIC according to the existence of PPH. PPH: Post Partum Hmorrhage. No PPH: All cases without Post partum Hemorrhage. ISTH: International Society on Thrombosis and Haemostasis. Data are number (%)The % values reported are those patients in whom the DIC could be measured. Patients who DIC could not be measured were excluded. NA: Not Applicable.
(DOCX)

S5 Table DIC according to the pregnancy complication. Data are N (%). DIC: disseminated intravascular coagulation. PPH: Post Partum Hemorrhage. PE: Preeclampsia. HELLP syndrome: HELLP syndrome: Hemolysis, Elevated Liver enzymes, Low platelet count Syndrome. AFLP: Acute Fatty Liver pregnancy.
(DOCX)
