# Supplementary material for: Comparison of Two Diagnostic Scores of Disseminated Intravascular Coagulation in Pregnant Women Admitted to the ICU
Source: PLoS One. 2016 Nov 18;11(11):e0166471. doi: 10.1371/journal.pone.0166471 (PMC5115738; doi:10.1371/journal.pone.0166471)
Supplement: S1 File — (DOCX) [file pone.0166471.s001.docx]

**Definitions:**

- Postpartum hemorrhage was diagnosed when blood loss in the first 24 hours after delivery was greater than 500 ml following vaginal delivery or 1000 ml following cesarean section [1].

- Preeclampsia was diagnosed if blood pressure was ≥ 140/90 mmHg and proteinuria ≥ 300 mg/day at anytime from week 20 of gestation [2]. HELLP syndrome was defined by the combination of thrombocytopenia (< 100 G/L), elevated liver enzymes (AST > 70 UI/L) and hemolysis. Hemolysis was defined by a bilirubin > 12 mg/L, LDH > 600 UI/L, haptoglobin < 0.5 mg/L, and presence of more than 0.5% schyzocytes. In patients who presented a postpartum hemorrhage and/or needed platelet transfusion, the decrease in platelet count was not used as diagnostic criteria of HELLP syndrome, except when the HELLP syndrome had clearly preceded the hemorrhagic complication [3].

- Criteria for diagnosis of acute fatty liver disease of pregnancy include six or more of the following (Swansea criteria): Vomiting, abdominal pain, polydipsia/polyuria, encephalopathy, elevated bilirubin (>14 μmol/L), hypoglycemia (<4 μmol/L), elevated blood uric acid (>340 μmol/L), leukocytosis (>11 × 10^9^/L), ascites or bright liver on ultrasound scan, elevated transaminases (aspartate aminotransferase or alanine aminotransferase >42 IU/l), elevated ammonia (>47 μmol/L), renal impairment (creatinine >150 μmol/L), coagulopathy (PT >14 seconds (s) or activated partial thromboplastin time >34 s [4]. The patients’ severity was assessed by the SAPS II score during the first 24 hours after admission in ICU [5].

**Criteria of DIC of the new score:**

This score uses three components: platelet count, fibrinogen concentration, and the PT difference compared to control. Specific thresholds have been determined from the ROC curves of each component. The specific weight of each parameter was calculated by logistic regression. A score ≥ 26 is considered consistent with the diagnosis of DIC[7].

**Criteria of DIC of the ISTH score**

The ISTH score can only be used in a determined clinical context known to be associated with DIC. It is based on the combination of biological variables: PT, platelets, fibrinogen, and one fibrinolysis marker (fibrin monomers, fibrin soluble complexes, D-dimers, or PDF). An ISTH score ≥5 is considered to reflect an overt DIC(8).

We used in our study either D-dimers or fibrin monomer (FM) assays with the following thresholds: a score 0 was given when D-dimers was < 0.5mg/l or FM < 6 mg/ml; a score 1 was given when D-dimers were between 0.5 and 10 mg/l or MF between 6 and 50 mg/ml; a score 2 was given when D-dimers were > 10 mg/l or MF > 50 mcg/ml

**References**

1. Subtil D, Sommé A, Ardiet E, Depret-Mosser S (2004) Postpartum hemorrhage: frequency, consequences in terms of health status, and risk factors before delivery. J Gynécologie Obstétrique Biol Reprod 33:4S9‑4S16.

2. ACOG practice bulletin (2002) Diagnosis and management of preeclampsia and eclampsia. Obstet Gynecol 99:159‑67.

3. Sibai BM (1990) The HELLP syndrome (hemolysis, elevated liver enzymes, and low platelets): much ado about nothing? Am J Obstet Gynecol 162:311‑6.

4. Knight M, Nelson-Piercy C, Kurinczuk JJ, Spark P, Brocklehurst P (2008) UK Obstetric Surveillance System. A prospective national study of acute fatty liver of pregnancy in the UK. Gut 57:951‑6.

5. Le Gall JR, Lemeshow S, Saulnier F (1993) A new Simplified Acute Physiology Score (SAPS II) based on a European/North American multicenter study. JAMA 270:2957‑6.

6. Erez O, Novack L, Beer-Weisel R, Dukler D, Press F, Zlotnik A, et al (2014) DIC Score in Pregnant Women – A Population Based Modification of the International Society on Thrombosis and Hemostasis Score. PLoS ONE. 9: e93240.

7. Taylor FB, Toh CH, Hoots WK, Wada H, Levi M (2001) Scientific Subcommittee on Disseminated Intravascular Coagulation (DIC) of the International Society on Thrombosis and Haemostasis (ISTH). Towards definition, clinical and laboratory criteria, and a scoring system for disseminated intravascular coagulation. Thromb Haemost 86:1327‑3 .
