# Supplementary material for: Comparison of Two Diagnostic Scores of Disseminated Intravascular Coagulation in Pregnant Women Admitted to the ICU
Source: PLoS One. 2016 Nov 18;11(11):e0166471. doi: 10.1371/journal.pone.0166471 (PMC5115738; doi:10.1371/journal.pone.0166471)
Supplement: S1 Fig — If score ≥ 26: compatible with DIC. (DOCX) [file pone.0166471.s002.docx]

S1 Fig. New DIC score by Erez.

|  | Assigned weight |
| --- | --- |
| PT difference (seconds) |  |
| < 0.5 | 0 |
| 0.5-1 | 5 |
| 1. - 1.5 | 12 |
| > 1.5 | 25 |
| Platelets (10^9^/L) |  |
| < 50 | 1 |
| 50-100 | 2 |
| 100- 185 | 1 |
| > 185 | 0 |
| Fibrinogen (g/L) |  |
| < 3.0 | 25 |
| 3.0- 4.0 | 6 |
| 4.0- 4.5 | 1 |
| > 4.5 | 0 |

If score ≥ 26 : compatible with DIC.
