# Supplementary material for: Comparison of Two Diagnostic Scores of Disseminated Intravascular Coagulation in Pregnant Women Admitted to the ICU
Source: PLoS One. 2016 Nov 18;11(11):e0166471. doi: 10.1371/journal.pone.0166471 (PMC5115738; doi:10.1371/journal.pone.0166471)
Supplement: S2 Fig — If ≥ 5: compatible with overt DIC: repeat score daily. If ≤ 5: suggestive (not affirmative) for non-overt DIC: repeat next 1–2 days. (DOCX) [file pone.0166471.s003.docx]

S2 Fig. ISTH DIC score.

|  | 0 | 1 | 2 | 3 |
| --- | --- | --- | --- | --- |
| Platelets (10^9^/L) | > 100 | < 100 | < 50 |  |
| Elevated fibrin related markers  (e.g. D-dimers, fibrin monomer, fibrin degradation products) | No increase |  | Moderate increase | Strong increase |
| Prolonged prothrombin time (seconds) | < 3 | > 3 but < 6 | > 6 |  |
| Fibrinogen (g/L) | > 1 | < 1 |  |  |

If ≥ 5: compatible with overt DIC: repeat score daily

If ≤ 5: suggestive (not affirmative) for non-overt DIC: repeat next 1-2 days.
