# Supplementary figures and images for: Comparison of Two Diagnostic Scores of Disseminated Intravascular Coagulation in Pregnant Women Admitted to the ICU
Source: PLoS One. 2016 Nov 18;11(11):e0166471. doi: 10.1371/journal.pone.0166471 (PMC5115738; doi:10.1371/journal.pone.0166471)

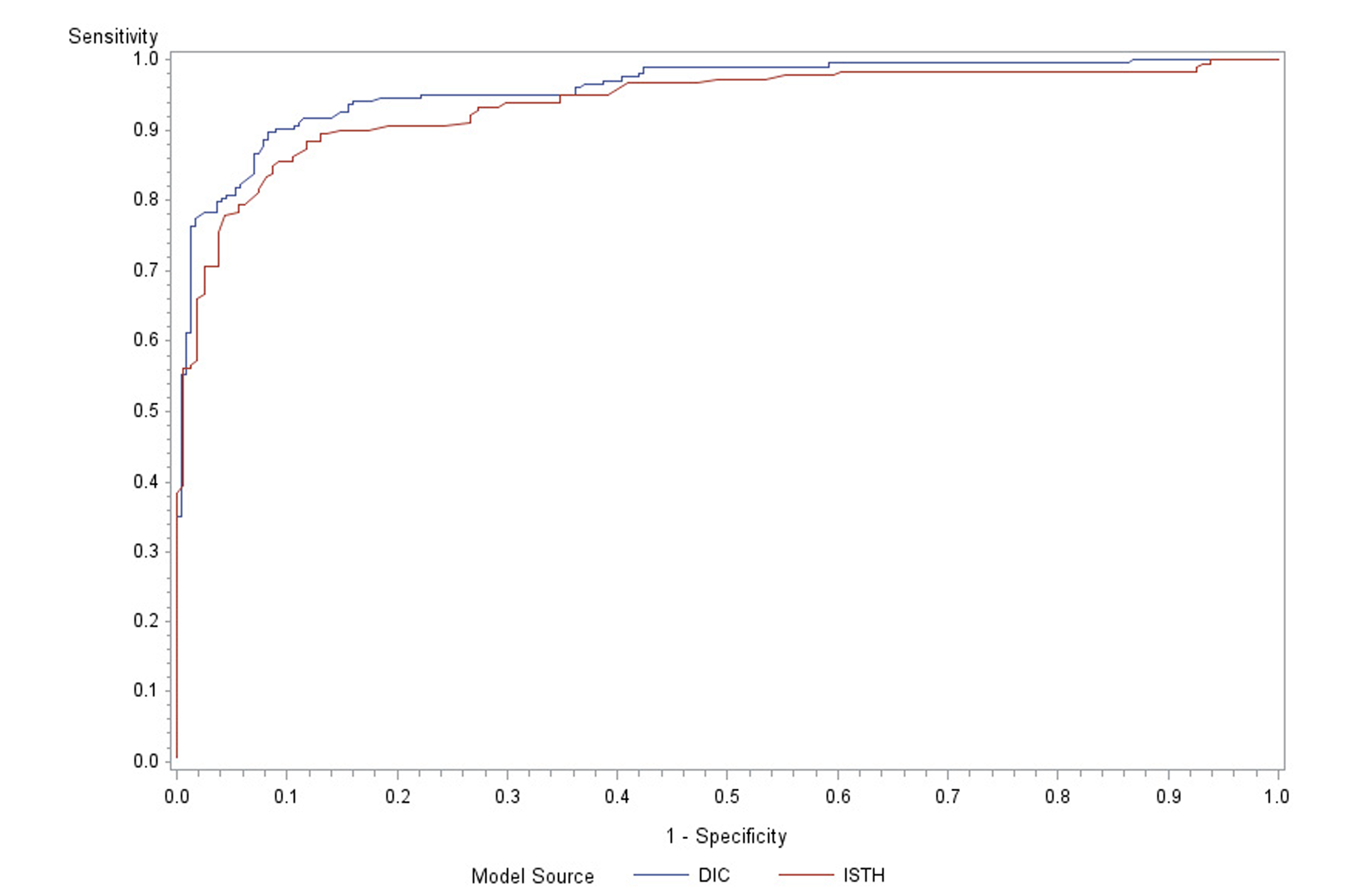

Supplement: S3 Fig — (TIFF) [file pone.0166471.s004.tiff]
