# Supplementary material for: Comparison of Two Diagnostic Scores of Disseminated Intravascular Coagulation in Pregnant Women Admitted to the ICU
Source: PLoS One. 2016 Nov 18;11(11):e0166471. doi: 10.1371/journal.pone.0166471 (PMC5115738; doi:10.1371/journal.pone.0166471)
Supplement: S1 Table — Data are N (%) for qualitative variables. * Quantitative variables are presented as median [interquartile range: 25th to 75th percentiles]. (DOCX) [file pone.0166471.s005.docx]

| **PPH** | n=85 |
| --- | --- |
| Isolated | 54 (64) |
| with PE | 6 (7) |
| with HELLP | 25 (29) |
| **Volume of bleeding *(ml)** | 3000 (1850-4475) |
| **Etiologies of PPH** |  |
| Uterine Atony | 25 (29) |
| Infectious | 8 (9) |
| PE/HELLP syndrome | 15 (17) |
| Abnormal placentation | 10 (12) |
| Placental abruption | 8 (9) |
| Vaginal laceration | 8 (9) |
| Retained placenta | 6 (7) |
| **Treatment** |  |
| Medical treatment | 21 (25) |
| Arterial ligation | 19 (22) |
| Arterial embolization | 8 (9) |
| Hysterectomy | 31(36) |
| others | 6 (7) |

**S1 Table.** Characteristics of patients with postpartum hemorrhage.

Data are N (%) for qualitative variables

^*^ Quantitative variables are presented as median [interquartile range: 25th to 75th percentiles]
