# Supplementary material for: Comparison of Two Diagnostic Scores of Disseminated Intravascular Coagulation in Pregnant Women Admitted to the ICU
Source: PLoS One. 2016 Nov 18;11(11):e0166471. doi: 10.1371/journal.pone.0166471 (PMC5115738; doi:10.1371/journal.pone.0166471)
Supplement: S2 Table — Data are N (%) for qualitative variables. (DOCX) [file pone.0166471.s006.docx]

**S2 Table.** Number and percentages of patients with DIC according to the two scores and expert analysis.

|  | **New score** | **ISTH** | **Expert analysis** |
| --- | --- | --- | --- |
| **Delivery** | 44 (28) | 16 (10) | 70 (45) |
| **Day 0** | 70 (45) | 25 (16) | 85 (55) |
| **Day 1** | 38 (24) | 11 (7) | 49 (32) |
| **Day 2** | 13 (8) | 5 (3) | 22 (14) |

Data are N (%) for qualitative variables
