# Supplementary material for: Comparison of Two Diagnostic Scores of Disseminated Intravascular Coagulation in Pregnant Women Admitted to the ICU
Source: PLoS One. 2016 Nov 18;11(11):e0166471. doi: 10.1371/journal.pone.0166471 (PMC5115738; doi:10.1371/journal.pone.0166471)
Supplement: S3 Table — Data are number; ISTH, International Society of Thrombosis and Haemostasis. (DOCX) [file pone.0166471.s007.docx]

|  | | **Delivery** | **Day 0** | **Day 1** | **Day 2** | **Global** |
| --- | --- | --- | --- | --- | --- | --- |
| **New score** | **ISTH score** |  |  |  |  |  |
| **+** | **+** | 14 | 23 | 10 | 3 | 50 |
| **-** | **+** | 2 | 2 | 1 | 2 | 7 |
| **+** | **-** | 21 | 42 | 21 | 7 | 91 |
| **-** | **-** | 17 | 60 | 66 | 50 | 193 |
| **Rate of concordance (%)** | | 57 | 65 | 77 | 85 | 71 |
| **Kappa Coefficient** | | 0.24 | 0.32 | 0.37 | 0.33 | 0.35 |

**S3 Table.** Agreement between the two scores.

Data are number ; ISTH, International Society of Thrombosis and Haemostasis
