# Supplementary material for: Comparison of Two Diagnostic Scores of Disseminated Intravascular Coagulation in Pregnant Women Admitted to the ICU
Source: PLoS One. 2016 Nov 18;11(11):e0166471. doi: 10.1371/journal.pone.0166471 (PMC5115738; doi:10.1371/journal.pone.0166471)
Supplement: S4 Table — PPH: Post Partum Hmorrhage. No PPH: All cases without Post partum Hemorrhage. ISTH: International Society on Thrombosis and Haemostasis. Data are number (%)The % values reported are those patients in whom the DIC could be measured. Patients who DIC could not be measured were excluded. NA: Not Applicable. (DOCX) [file pone.0166471.s008.docx]

| DIC | | PPH | No PPH | p |
| --- | --- | --- | --- | --- |
| Expert analysis | | | | |
| Delivery (n=70) | | 53 (79) | 17 (39) | <0.01 |
| Day 0 (n=85) | | 59 (69) | 26 (39) | <0.01 |
| Day 1 (n=49) | | 33 (41) | 16 (25) | 0.04 |
| Day 2 (n=22) | | 12 (20) | 10 (22) | NA |
| Global (n=226) | | 157 (53) | 69 (31) | <0.01 |
| New score | | | | |
| Delivery (n=44) | | 37 (80) | 7 (30) | <0.01 |
| Day 0 (n=70) | | 52 (68) | 18 (27) | <0.01 |
| Day 1 (n=38) | | 25 (35) | 13 (21) | NA |
| Day 2 (n=13) | | 5 (9) | 8 (18) | NA |
| Global (n=165) | | 119 (47) | 46 (23) | <0.01 |
| ISTH | | | | |
| Delivery (n=16) | 13 (37) | | 3 (16) | NA |
| Day 0 (n=25) | 16 (24) | | 9 (15) | NA |
| Day 1 (n=11) | 7 (13) | | 4 (9) | NA |
| Day 2 (n=5) | 2 (6) | | 3 (10) | NA |
| Global n=57 | 38 (20) | | 19 (12) | NA |

**S4 Table** : Comparison of the rate of DIC according to the existence of PPH.

PPH : Post Partum Hmorrhage

No PPH : All cases without Post partum Hemorrhage

ISTH: International Society on Thrombosis and Haemostasis

Data are number (%)The % values reported are those patients in whom the DIC could be measured. Patients who DIC could not be measured were excluded

NA : Not Applicable
