# Supplementary material for: Comparison of Two Diagnostic Scores of Disseminated Intravascular Coagulation in Pregnant Women Admitted to the ICU
Source: PLoS One. 2016 Nov 18;11(11):e0166471. doi: 10.1371/journal.pone.0166471 (PMC5115738; doi:10.1371/journal.pone.0166471)
Supplement: S5 Table — Data are N (%). DIC: disseminated intravascular coagulation. PPH: Post Partum Hemorrhage. PE: Preeclampsia. HELLP syndrome: HELLP syndrome: Hemolysis, Elevated Liver enzymes, Low platelet count Syndrome. AFLP: Acute Fatty Liver pregnancy. (DOCX) [file pone.0166471.s009.docx]

|  | **Delivery** | **Day 0** | **Day 1** | **Day 2** |
| --- | --- | --- | --- | --- |
| **AFLP (n=6)** |  |  |  |  |
| Expert analysis | 4 | 6 (100) | 6(100) | 6(100) |
| New score | - | 6(100) | 6(100) | 6(100) |
| ISTH | - | 4(67) | 4(67) | 3(50) |
| **HELLP SYNDROME (n= 61)** |  |  |  |  |
| **HELLP without PPH (n=36)** |  |  |  |  |
| Expert analysis | 8 (22) | 12 (33) | 8 (22) | 2 (5) |
| New score | 4 (11) | 8 (22) | 5 (14) | 1 (3) |
| ISTH | 3 (8) | 5 (14) | 0 | 0 |
| **HELLP with PPH (n=25)** |  |  |  |  |
| Expert analysis | 14 (56) | 21 (84) | 13 (52) | 7 (28) |
| New score | 12 (48) | 18 (72) | 11 (44) | 3 (12) |
| ISTH | 6 (24) | 8 (32) | 3 (12) | 2 (8) |
| **PE n=27** |  |  |  |  |
| **PE without PPH n=21** |  |  |  |  |
| Expert analysis | 4 (19) | 7 (33) | 2 (9) | 2 (9) |
| New score | 2 (9) | 3 (14) | 2 (9) | 1 (5) |
| ISTH | 0 | 0 | 0 | 0 |
| **PE with PPH n=6** |  |  |  |  |
| Expert analysis | 2 (33) | 4 (67) | 1 (17) | 1 (17) |
| New score | 1 (17) | 2 (33) | 0 | 0 |
| ISTH | 0 | 0 | 0 | 0 |
| **PPH alone (n=54)** |  |  |  |  |
| Expert analysis | 37 (68) | 34 (63) | 19 (35) | 4 (7) |
| New score | 24 (44) | 32 (59) | 14 (26) | 2 (4) |
| ISTH | 7 (13) | 8 (15) | 4 (7) | 0 |

**S5 Table**: DIC according to the pregnancy complication.

Data are N (%)

DIC: disseminated intravascular coagulation

PPH : Post Partum Hemorrhage

PE : Preeclampsia

HELLP syndrome: HELLP syndrome: Hemolysis, Elevated Liver enzymes, Low platelet count Syndrome

AFLP : Acute Fatty Liver pregnancy
